# Healthcare personnel’s perspectives on health technology in home-based pediatric palliative care: a qualitative study

**DOI:** 10.1186/s12904-024-01464-w

**Published:** 2024-05-29

**Authors:** Judith Schröder, Kirsti Riiser, Heidi Holmen

**Affiliations:** 1 Faculty of Health Sciences, Department of Nursing and Health Promotion, Oslo Metropolitan University, St. Olavs plass, P.O. Box 4, Oslo, NO-0130 Norway; 2 Faculty of Health Sciences, Department of Rehabilitation Science and Health Technology, Oslo Metropolitan University, St. Olavs plass, P.O. Box 4, Oslo, NO-0130 Norway; 3 Division of Technology and Innovation, Intervention Centre, Oslo University Hospital, Oslo, Norway

**Keywords:** Pediatric palliative care, Healthcare personnel, Home-based, Ehealth, Digital health

## Abstract

**Background:**

In the context of pediatric palliative care, where the quality of life of children with life-limiting or life-threatening conditions is of utmost importance, the integration of health technology must support the provision of care. Research has highlighted the role of healthcare personnel when utilizing health technology in home-based pediatric palliative care, but specific knowledge of healthcare personnel’s views on the technological relevance remains limited. Therefore, our study has explored potentials and limitations of health technology in home-based pediatric palliative care from the perspectives of healthcare personnel.

**Methods:**

Our study utilized a qualitative, descriptive, and exploratory design, including five focus groups with a total of 22 healthcare personnel. The participants were selected from various health regions in Norway and were experienced in providing home-based pediatric palliative care. Using reflexive thematic analysis, we interpreted data obtained from focus groups, identified patterns, and developed themes.

**Results:**

The analysis resulted in the development of three intersecting themes: *balancing in-person interaction and time in home-based pediatric palliative care; exchange of information can improve timely and appropriate care; and the power of visual documentation in pediatric palliative care.* The healthcare personnel acknowledged difficulties in fully replacing in-person interaction with health technology. However, they also emphasized potentials of health technology to facilitate information sharing and the ability to access a child’s health record within interdisciplinary teams.

**Conclusion:**

The results underscored that technology can support pediatric palliative care but must be thoughtfully integrated to ensure an individualized patient-centered approach. To maximize the benefits of health technology in enhancing home-based pediatric palliative care, future research should address the limitations of current health technology and consider the opinions for information sharing between relevant healthcare team members, the child, and their family.

**Supplementary Information:**

The online version contains supplementary material available at 10.1186/s12904-024-01464-w.

## Background

Pediatric palliative care focuses on maximizing the quality of life for children with life-limiting or life-threatening conditions and their families [1], with home-based care aiming to provide a sense of normalcy based on their preferences [1–5]. One way of supporting home-based care is using health technology, which has been suggested to reduce traveling, enhance communication between families and healthcare personnel, and enable peer-to-peer support from pediatric specialists in hospitals for home-care teams [3–7]. However, there are various understandings and definitions of what health technology entails [3–7]. In our study, health technology encompasses the broad utilization of information and communication technologies to enhance patient-centered care, including eHealth [4], telehealth [5], telemedicine [3], or digital health [6], hence adopting a comprehensive approach.

One important aim of health technology in home-based pediatric palliative care is to enhance the accessibility of specialized pediatric palliative care [3–7]. Previous research on health technology in palliative care comprises videoconferencing, remote patient monitoring for assessment, mobile applications for information sharing, and electronic health records accessible in different healthcare settings [8, 9]. However, research has predominantly focused on using these tools in palliative care for adults, with limited exploration in pediatric palliative care [3–7]. In pediatric palliative care, videoconferencing has gained the most attention because it is suggested to facilitate consultations, allow for valuable face-to-face interactions, and promote individualized patient-centered care [3–7, 10, 11]. Furthermore, videoconferencing can be useful for healthcare personnel for effective care team meetings, peer-to-peer support, and education [4, 6]. However, videoconferencing is often used as a supplementary tool rather than a complete substitute for in-person meetings [3–6, 10, 11]. Various factors have been reported to hinder the widespread adoption of videoconferencing among healthcare personnel. These include hesitations towards engaging in sensitive conversations over video and concerns about privacy [3–6]. Moreover, when multiple members of the care team participate in the same videoconference it can create an overload of excessive healthcare-related information for families, which could potentially lead to over-servicing and risk overwhelming families [10, 11]. Furthermore, research suggests that healthcare personnel might require time to acclimate to using video technology in clinical practice and may experience a lack of control when working with unfamiliar and uncomfortable technological interfaces [11]. Finally, healthcare personnel expressed concerns about potential impacts on the relationship they build with children and their families, and they worried that videoconferences might impede, disrupt, or alter this crucial relationship [4, 5, 10, 11]. These hesitations compromise the potential efficiency and cost-saving benefits of videoconferencing [3–6].

Research emphasizes that healthcare personnel play a crucial role in implementing health technologies in home-based pediatric palliative care [3–6]. If healthcare personnel encounter challenges or hold negative attitudes toward health technology, it may lead to reduced acceptance and less utilization in clinical practice [10, 11]. However, there is limited research available addressing the use of health technology by healthcare personnel in home-based pediatric palliative care [3–6]. Our study examined healthcare personnel’s perspectives on using health technology in this context in Norway, aiming to explore its potentials and limitations.

## Methods

Our study employed a qualitative descriptive and exploratory approach, giving comprehensive insights into healthcare personnel’s perspectives through focus groups.

### Setting, recruitment, participants

The publicly funded Norwegian healthcare system offers comprehensive and free coverage to all residents, with primary healthcare services being organized and managed by municipalities [12]. Efforts have been made to establish a comprehensive children’s palliative care service, supported by national guidelines published by the Norwegian Directorate of Health in 2016 [13]. The commitment to delivering healthcare services for seriously ill children also finds prominence in the government’s digitalization strategy for the 2019–2025 period [14], emphasizing the importance of providing citizens with a user-friendly and seamless experience through utilization and exchange of data within a collaborative digital ecosystem. The use of health technology in Norway, as in many other countries, is implemented to help enhance and streamline home-based healthcare services [14]. The use of health technology can address important aspects, including the efficient utilization of resources, financial sustainability, and the environmental impact of healthcare services [12]. Additionally, health technology can enable timely care delivery to rural and remote populations [15].

We applied purposive sampling, targeting healthcare personnel involved in primary healthcare services in Norway with valuable expertise in home-based pediatric palliative care [16]. The first author JS obtained contact information of healthcare personnel through three sources: the advisory group of the research project “Children in Palliative Care - health technology in home-based pediatric palliative care” (CHIP homeTec) [17], and pediatric palliative care teams at two university hospitals. Eight municipal healthcare services were contacted and invited to receive information about the study design by phone and email. Of these, five municipal services accepted their participation in the study, while three declined due to heavy workloads.

The participants were healthcare personnel employed in primary healthcare services that were located across different regions in Norway. These primary healthcare services served populations ranging from 3,750 to 77,550 inhabitants and encompassed areas from 70 to 2,500 square kilometers. The jurisdictions of these primary healthcare services included both urban and rural areas. Five focus groups were created with 22 participants. One group included three participants, another had four participants, and the remaining three groups had five participants each. One participant was male. Table 1 presents the self-reported professions of study participants and their respective years of experience in pediatric palliative care. The participants included physiotherapists (*n* = 8), occupational therapists (*n* = 6), public health nurses (*n* = 2), child and youth workers (*n* = 2), assistant nurses (*n* = 2), one palliative care nurse, and one social worker. All participants were providing primary healthcare services to children in general and specifically to children with palliative care needs. The participants’ work experience in pediatric palliative care ranged from 6 months to 30 years, with an overall median duration of 11 years.

**Table 1:** Self-reported Participant Characteristics

Survey item	*N* = 22
Participants profession	
Physiotherapists	8
Occupational therapists	6
Public health nurses	2
Child and youth workers	2
Assistant nurses	2
Palliative care nurse	1
Social worker	1
Participants pediatric palliative care experience	
0–1 year	1
1–5 years	5
6–10 years	5
11–20 years	8
> 21 years	3

### Data collection

Before participating in the focus groups, all participants completed a web-based questionnaire regarding their profession, workplace, and years of experience in pediatric palliative care. The Service for Sensitive Data (TSD) provided by the University of Oslo was utilized to collect and store all data from the questionnaires. The TSD server is specifically designed to ensure data security [18].

We conducted focus groups by following established guidelines [19]. A pilot focus group was conducted to test the brief and flexible semistructured interview guide and the audio recording tool. The interview guide was designed to be broad and flexible, stimulating wide-ranging discussions on the use of health technology by healthcare personnel with minimal steering from the moderator [20]. Starting with open-ended questions about general experiences in pediatric palliative care often led participants to relate to positive experiences and what was working well for them. To facilitate a balanced conversation, we incorporated specific prompts addressing barriers and limitations. Additionally, conversations around desired innovations for streamlining home-based pediatric palliative care naturally highlighted the potential benefits of health technology. The complete interview guide in English language is available as supplementary material 1.

JS (moderator) and KR or HH (secretary) met the respondents for the first time at the designated focus group. At the fourth focus group, another Ph.D. candidate filled in as the secretary. The focus groups were conducted during working hours between March and April 2023 at the participants’ workplaces. The purpose of the research project, the background and research interests of the researchers, and the roles of the moderator and secretary were presented to the participants before the interviews started. A brief introduction to health technology as communication and information technology used in remote care was given. The interviews were recorded digitally, and the recordings were stored and transcribed onto the TSD server [18]. Three of the focus groups were transcribed by JS, and two were transcribed using Whisper, a speech recognition model that transcribes audio files [21]. JS ensured the accuracy of the transcriptions produced by Whisper by thoroughly reviewing and editing them multiple times. All transcriptions were edited and adapted to written language norms. The focus groups lasted between 63 and 87 min.

### Analysis

We employed reflexive thematic analysis, which offers theoretical flexibility and systematic guidelines for a comprehensive exploration of the dataset [22]. The analysis followed a constructivist paradigm, aiming to understand the meaning that the participants attributed to their perspectives on health technology in home-based pediatric palliative care.

JS analyzed the data by repeatedly listening to the recorded discussions and reviewing the transcripts to become familiar with the data. KR and HH were given access to the transcripts, and we met to exchange our initial thoughts and reflections on the dataset.

Progressing to the second phase of generating the initial codes [22], we conducted an inductive approach to best represent meaning as communicated by the participants and identify relevant sections of data within the dataset; we then subsequently categorized similar codes and their associated meanings (Table 2). In phase three, we developed provisional candidate themes on shared meaning by grouping codes [22]. This process was not strictly linear because we switched between phases three and four, where themes were continuously re-evaluated to uncover underlying contradictions and deeper insights into the participants’ perspectives. We were cognizant of our role as cocreators of themes and approached the interpretation of data through the lens of our perceptions and understanding of previous research [22]. JS prepared a summary for each theme during phase five and shared these summaries with KR and HH. Together, we worked collaboratively to enhance, clarify, and label the identified themes. In our analysis, we concentrated on identifying the main themes that provided a detailed, interpretive, and contextualized narrative of our dataset [22]. As a result, we did not establish any subthemes. Finally, JS created an analytical narrative that addressed the research question, which was reviewed and edited by KR and HH for accuracy and clarity.

**Table 2:** Examples to demonstrate the coding process, conducted sentence-by-sentence

Transcribed data	Codes
My initial thought is that technology can never replace all the care from healthcare personnel, no matter what _(C1)_.	(C1) Technology cannot replace care from healthcare personnel.
It is not either/or, and that is probably not the goal either, but rather that one must discuss when to use it (*technology*) _(C2)_.	(C2) The use of technology needs to be discussed.
We, as physiotherapists and occupational therapists, provide some tasks that technology cannot completely replace _(C3)_.	(C3) Healthcare personnel’s work cannot be replaced by technology.
It could be that incorporating other professionals who are not as accessible can be possible through technology, such as video calls _(C4)_.	(C4) Technology can facilitate contact with healthcare personnel who are unavailable.
Perhaps as physiotherapists or occupational therapists, we can physically be in the child’s home while connecting with a social worker or doctor who may not be as accessible _(C5)_.	(C5) Healthcare personnel at the child’s home can connect with other remote healthcare personnel.

### Preunderstanding

All authors (JS, KR, HH) are actively engage in the CHIP homeTec research project, which investigates the utilization of health technology in home-based pediatric palliative care [17]. Our preunderstanding has been influenced by our previous systematic review. The results from this review highlighted the needs of healthcare personnel when delivering home-based pediatric palliative care. These needs encompass the establishment of relationships with both the child and their family, effective collaboration within the healthcare team, and the provision of services in an environment that guarantees fairness and long-term sustainability [23].

### Ethical considerations

Our study was approved by the Norwegian Agency for Shared Services in Education and Research, which concluded that our study was in accordance with the Personal Data Act (reference number 657413). Because our study solely involved healthcare personnel and did not collect health data, it did not require permission from a regional committee for research ethics. Prior to participating in the focus groups, all individuals were extensively informed, both verbally and through written communication, about the voluntary nature of their involvement, along with the assurance of anonymity, confidentiality, and option to withdraw from the study without providing any reasons. Written informed consent was obtained from all participants before their participation in the focus groups.

## Results

The focus group participants reflected on the potentials and limitations of health technology by referring to existing health technologies in their practice, the experiences they acquired during the COVID-19 pandemic, and how they used social media in everyday life. This combination of informal and formal experience with health technology, combined with the many years of experience in home-based pediatric palliative care, gave the participants a broad foundation for discussions. The participants in all groups demonstrated an understanding of pediatric palliative care as a service that offers care, comfort, and emotional support while aiming to improve the quality of life for children with life-limiting or life-threatening conditions and their families.

The participants commonly utilized videoconferencing and electronic patient records as health technology solutions in their work. Additionally, some participants had experience with software applications specifically designed for children with cognitive impairments and limited language ability, as well as software applications used to encourage physical activity.

The analysis resulted in the development of three intersecting themes: *balancing in-person interaction and time in home-based pediatric palliative care; exchange of information can improve timely and appropriate care; and the power of visual documentation in pediatric palliative care*.

### Balancing in-person interaction and time in pediatric palliative care

The participants emphasized that, within pediatric palliative care, health technology must be employed with caution and should be guided by a clear purpose that weighs the benefits of use against possible distress or harm to the family. The participants deemed it essential to emphasize that, although health technology can serve as a helpful supplement, it could never fully replace the unique competence and invaluable human connection that healthcare personnel provide to the families they serve. They emphasized a sensitive and holistic approach to care, relying on in-person interactions and strong relationships built on trust and empathy as critical aspects that technology cannot replicate.

*The personal meetings, which I think are enormously important, should not be fully replaced (by technology)*. (Occupational therapist, group 3)

Some participants added that in-person interaction in the children’s homes can provide a valuable break for families dealing with exhausting situations and, therefore, can have a positive impact on the quality of life of both the child and family.

The participants also highlighted the therapeutic value of hands-on evaluation, where healthcare personnel apply their many years of experience and expertise to assess the child’s condition, an expertise that health technology cannot replicate. They further argued that, the utilization of health technology falls short of conveying the essential nonverbal cues related to the child’s well-being.

*It can be a small tension in the child’s body that you interpret as something not quite right. Can we try to figure out what it is? A sigh that lasts too long, or an expiration that doesn’t come where you expect that it should. What is it? It is much easier to act as a therapist when you can be physically present.* (Physiotherapist, group 5)

The participants were concerned that current health technologies can be burdensome and time-consuming, which takes time away from caring for children and their families. They highlighted specific issues with current electronic medical records and messaging platforms as examples of suboptimal technologies. Their experiences revealed several challenges, such as a lack of integration between different systems, the annoyance of two-factor authentication, connectivity problems with portable devices, and character limits in existing messaging platforms.

*There is only a maximum number of characters you can write. (…). And then I have to write four messages because I have to complete what I have to say. And then I have to divide it into four parts. In fact, the system should make everyone’s everyday life more efficient. But it doesn’t because it’s not good enough.* (Occupational therapist, group 3)

The citation above illustrates the trouble healthcare personnel faced when trying to use a messaging platform to communicate with parents and serves as an example of how health technology hinders—rather than helps—in providing comprehensive palliative care.

Although in-person interactions were deemed critical, the participants discussed situations in which health technology could be particularly useful. One such example was when healthcare personnel have a cold and there is a risk of infecting the child and family during a home visit, using technology could be a practical alternative instead of canceling the visit.

### Exchange of information can improve timely and appropriate care

The participants also discussed the crucial aspects of interdisciplinary collaboration in pediatric palliative care. Although the multidisciplinary nature of the palliative care team was acknowledged as highly beneficial for the child and family, it posed a challenge for the participants to establish efficient collaboration. Ensuring they had the latest updates on the child’s status before home visits was a concern because of the many professionals involved in care delivery and the often-unpredictable illness trajectory of the children. Communication and information exchange between parents, healthcare personnel, educational staff, and personal assistants was viewed as critical. However, the participants recognized a lack of tools that could facilitate effective information flow, which resulted in frequent use of traditional communication methods such as phone calls and emails. These practices were not only reported to be time-consuming but also raised concerns regarding the protection of confidentiality for the child. The participants pointed out that the obstacles to information exchange could lead to delays in necessary care, potentially affecting outcomes for children and their families.

To overcome care fragmentation and promote seamless exchange of information, the participants envisioned one shared digital platform to serve as a central source of information. This platform should allow all care team members access to the child’s health data, daily reports, and advance care plan, regardless of their location or the time of day. As a result, healthcare personnel might be informed and better prepared for home visits.

*In situations where there are multiple people involved and handovers are necessary, one shared platform would be beneficial. It would allow for better preparation and understanding of what to expect before meeting the child and family in their home.* (Public nurse, Group 1)

The participants progressed in their dialogue by contemplating strategies for how to engage healthcare, educational, and welfare professionals, all of whom deliver services to children and families in their homes, in the exchange of information. They highlighted the potential of one shared digital platform that could bring the various professions together.

Some participants said that they only needed access to crucial information relevant to their work rather than all kinds of information. Others feared accountability for tasks that they might not grasp thoroughly from their professional perspective, emphasizing the need for a comprehensive dialogue before task allocation.

Furthermore, the participants emphasized the potential benefits of features such as chat messages and videoconferencing, underscoring the importance of direct verbal information.

*In many cases, what could have been highly beneficial is a group chat. A straightforward, old-fashioned group chat. So much information comes through different channels.* (Physiotherapist, group 5)

Communication technologies were praised for their ability to improve meeting efficiency by uniting all care team members, regardless of their location, promoting timely and effective information sharing across the team, and providing easy access to peer support. The participants also recognized the potential of these functionalities to alleviate burdensome traveling for the children and their families.

Some participants suggested that parents should have access to the shared digital platform as well, considering their role as the child’s primary caregivers and important members of the care team. However, others acknowledged that parents could feel overwhelmed with excessive information and that healthcare personnel would hesitate to share all the details with the parents. Furthermore, some participants requested communication technologies that resonate with and engage young people. They emphasized the importance of interactive platforms that enable direct communication with adolescents, free from parental interference, for the exchange of relevant information.

### The power of visual documentation in pediatric palliative care

An important aspect consistently discussed across all focus groups was the possibility of supplementing written documentation with photos and videos to facilitate understanding of the child’s health condition in both acute cases and over time. The participants recognized pediatric palliative care as a prolonged duration of care characterized by a complex progression of the child’s condition. The participants suggested that visual documentation could provide more comprehensive and factual firsthand information. This could help reduce the element of personal interpretation and the potential errors that can commonly occur in unspecific written reports. Visual documentation was suggested to ensure accuracy in assessing and managing the child’s condition, function, and progression. The importance of visual documentation was highlighted as particularly relevant in communication with hospital staff, which tended to rely on observations from home care personnel to reassess ongoing treatments.

*Do you trust yourself in everything you see? There are also variations from day to day and week to week. So, there’s something about getting that documented for quality assurance. There will be an interpretation anyway, but still, then, several are involved in that interpretation.* (Physiotherapist, group 1)

Two focus groups shared their experiences with a software application specifically designed for children with cognitive impairments and limited or no language ability [24]. This application enabled participants to document information using videos, photos, and textual descriptions.

*You get to know the child through the screen in a way, and can see how you do it, when the child is to be lifted into a chair. What does it mean when a child makes this sound or this facial expression? A way for me to get to know the child is through this app*. (Child and youth worker, group 2)

The participants discussed, based on their experiences, how visual documentation played a vital role in maintaining care, information exchange, and transitions during personnel shifts as well as being a valuable training resource, particularly for training assistants on the proper utilization of specialized equipment.

## Discussion

Our study aimed to explore healthcare personnel’s perspectives on the potential and limitations of health technology in home-based pediatric palliative care. The themes have underscored that technology can support care but must be carefully integrated to ensure that it adds to the individualized patient-centered approach in pediatric palliative care. Additionally, our analysis emphasized the potential of one shared digital platform that can store and exchange pertinent healthcare information, including multimedia files, enabling healthcare personnel to deliver timely and appropriate home-based pediatric palliative care.

The participants emphasized that pediatric palliative care aims to meet the comprehensive needs of both the child and the family. Our results corresponded with previous research describing that the use of health technology should be balanced with in-person interaction [3–6]. However, a systematic review explored how hands-on practitioners adapted to web-based formats during the COVID-19 pandemic and found various methods to overcome the lack of physical closeness [25]. The methods used by practitioners included improved communication skills for clear instructions, using visual aids like dolls, invoking sensorial memories, utilizing additional tools for engagement, incorporating music and singing, sending materials to participants’ homes, and encouraging the use of emojis or participation in polls [25]. Nevertheless, the successful implementation of these methods necessitated practitioners dedicating sufficient time for preparation, including briefing individuals who may assume their role in the child’s home as well as ensuring clear communication and setting expectations for effective web-based consultations [25]. This can be challenging given the time constraints already faced by healthcare personnel in home-based pediatric palliative care. While health technology can offer accessible and time-saving services, particularly for patients living in rural areas with limited access to healthcare resources or facilities [25], our results also showed that health technology may disrupt or change established practices that place a strong emphasis on in-person interaction and assessment. Future research should incorporate co-design principles for developing health technology [6] or focus on specific evaluations aligned with the assessment of the child’s condition [5]. Additionally, addressing healthcare personnel’s knowledge and perceptions may enhance the adaptation and utilization of health technology in home-based pediatric palliative care [4].

The desire for one shared digital platform for exchanging information across health services was expressed in all focus groups. In this regard, our study can offer unique insights into the potential of health technology, expanding beyond existing research that has primarily focused on videoconferencing [3–6, 10, 11]. The idea that all participants in the healthcare team can be “connected” by sharing and presenting timely, accurate, and relevant information about the child’s health is in line with Caulfield and Donnelly’s connected health model [26]. Caulfield and Donnelly underlined the value of information exchange in enhancing care models and introduced the connected health model as a sociotechnical approach that links people, processes, and technology through more intelligent utilization of data, devices, and communication platforms [26–28]. Research in palliative care has emphasized the information exchange value of electronic health records available across healthcare settings because information exchange improves continuity of care, ensures that patients are treated in line with their wishes, and streamlines clinical workflow [9, 29, 30].

One shared digital platform for the exchange of information about a child’s health and well-being in pediatric palliative care must be user-friendly and functional to be clinically relevant [29, 30]. Usability can be secured either by integrating the platform with existing electronic health record systems or replacing current health record systems altogether. Structuring documentation by using templates, order sets, and prompts has been suggested to alleviate the challenge of locating pertinent information [9, 29, 30]. However, previous research has suggested that healthcare personnel can encounter difficulties in rapidly accessing relevant information within electronic health records in the context of palliative care [29]. The often-long time frame of pediatric palliative care services, combined with the complex interdisciplinary team documentation, may challenge the organization of information within one shared digital platform. Our results underscored the need for user-friendly and functional technology that minimizes the time burden placed on healthcare personnel. Using health technology as a tool to enhance the efficiency of information exchange has also been highlighted as a core potential in previous research [29, 30].

In considering user-friendly and functional health technology, it is crucial to evaluate how well these technologies fulfill the specific requirements and needs of healthcare personnel who provide home-based pediatric palliative care [6, 31]. The assumption that health technology can effectively address resource efficiency, financial sustainability, and reduced environmental impact of healthcare services [32] has highlighted the increasing array of health technologies expected to be implemented in the future. Given the dynamic nature of palliative care, regular evaluations are necessary to ensure that current health technology solutions continue to meet the evolving needs of healthcare personnel [6, 31]. Engaging healthcare personnel in the evaluation and design process can help improve the usability and functionality of health technology and prevent it from adding additional strain to healthcare services; this involves actively seeking out healthcare personnel’s insights and perspectives to optimize current technology solutions or collaboratively design new ones that effectively support their work tasks [6, 31].

Our study uncovered contrasting viewpoints among the participants about whether parents should have access to the suggested shared digital platform. The potential to involve patients in managing their health has also been acknowledged in Caulfield and Donnelly’s connected health model [26]. Previous research has been limited regarding the experiences of children, parents, and healthcare personnel when sharing day-to-day information and evaluations. However, a systematic review highlighted how parents in pediatric palliative care report a greater sense of control over their family’s care at home and appreciate the ability to stay connected with healthcare personnel in the hospital [4]. It is important to acknowledge the fundamental importance of parents throughout their children’s lives in terms of communication and decision-making [33]. As time progresses, most parents develop unique skills in caring for their children, managing symptoms, and administering medication [2]. Thus, parents become valuable sources of information about their children’s care needs. Enabling access to one shared digital platform can enhance care coordination and ensure a family-centered approach. However, the families of children with palliative care needs often face challenges in terms of time management and responsibilities [33]. Introducing health technology may bring about additional complexities and demands for these caregivers [4]. Therefore, it is crucial to acknowledge and prioritize the preferences and needs of each family when considering the provision of access to health information, regardless of the use of health technology. An ideal would be to provide access to one shared digital platform as an optional feature tailored to the specific requirements and preferences of individual families, which is in line with established standards for pediatric palliative care [1].

In our study, the participants also highlighted the importance of health technology enabling direct communication with children, independent of parental involvement. Today children may feel more comfortable using digital solutions to interact with healthcare personnel. Therefore, research need to explore how health technology can enhance communication and interaction with children in the context of home-based pediatric palliative care. Allowing children to digitally report their health outcomes, can actively involve them in decisions regarding their care and treatment [4, 34].

### Strengths and limitations

We (JS, KR, HH) have varied clinical experiences in healthcare services which might led us to not prompt participants for further elaboration on their perspectives. However, our expertise with qualitative research methods helped us remain aware that we should not take the participants’ perspectives for granted. Moreover, the focus groups were conducted with a moderator and a secretary, allowing for an effective division of responsibilities, and reducing the risk of missing important perspectives. The focus groups were conducted in different health regions of Norway, ensuring geographical spread and greater variation in the data.

The focus groups primarily included physiotherapists and occupational therapists with their respective work responsibilities. The distinct roles and duties they held potentially restricted the transferability of the results to other professions like nurses or physicians. It is possible that nurses and physicians would have presented different or supplementary perspectives, thereby enhancing the discussions within the focus groups. However, one focus group did not include physiotherapists or occupational therapists, and the topics discussed were similar to those raised in the other groups. In discussing health technology in a general sense, the participants might have overlooked nuances and specificity related to the implementation and integration of specific health technology. However, the themes developed in this study encompassed both well-known aspects of implementing health technology and novel insights, indicating a substantial variation in the dataset.

## Conclusion

Our results showed that health technology in home-based pediatric palliative care can present both potential and limitations. Health technology has the potential to improve information sharing and, in turn, strengthen connections within the care team, leading to more proactive and efficient home-based pediatric palliative care. However, it is essential to recognize that close relationship and in-person interaction with the child is crucial in home-based pediatric palliative care and cannot be replaced by technology. Furthermore, there is a need for continuous evaluation to ensure that the health technology being used is effective, reliable, and suitable for the unique nature of home-based pediatric palliative care. Continuous evaluation of health technology is necessary to ensure the specific needs of healthcare personnel providing home-based pediatric palliative care. Future research should address the limitations of current health technology and consider the opinions for information sharing between all parties to maximize the benefits of health technology in enhancing home-based pediatric palliative care.

### Electronic supplementary material

Below is the link to the electronic supplementary material.

Supplementary Material 1

## Data Availability

The data transcripts generated and analyzed during our study are not publicly available because of legal restrictions. However, interested individuals may request access to the data from the authors. It is important to note that obtaining the data will require a reasonable request and permission from the Norwegian Agency for Shared Services in Education and Research.

## References

[1] Benini F, Papadatou D, Bernadá M, Craig F, De Zen L, Downing J (2022). International standards for Pediatric Palliative Care: from IMPaCCT to GO-PPaCS. J Pain Symptom Manage.

[2] Malcolm C, Knighting K, Taylor C (2020). Home-based end of Life Care for Children and their families – A systematic scoping review and narrative synthesis. J Pediatr Nurs.

[3] Miller KA, Baird J, Lira J, Herrera Eguizabal J, Fei S, Kysh L (2021). The Use of Telemedicine for Home-based Palliative Care for Children with Serious Illness: a scoping review. J Pain Symptom Manag.

[4] Holmen H, Riiser K, Winger A (2020). Home-based Pediatric Palliative Care and Electronic Health: systematic mixed methods review. J Med Internet Res.

[5] Bradford N, Armfield NR, Young J, Smith AC (2013). The case for home based telehealth in pediatric palliative care: a systematic review. BMC Palliat Care.

[6] Bird M, Li L, Ouellette C, Hopkins K, McGillion MH, Carter N (2019). Use of synchronous digital health technologies for the care of children with special health care needs and their families: scoping review. JMIR Pediatr Parent.

[7] Delemere E, Maguire R (2021). The role of Connected Health technologies in supporting families affected by paediatric cancer: a systematic review. Psychooncology.

[8] Disalvo D, Agar M, Caplan G, Murtagh FEM, Luckett T, Heneka N (2021). Virtual models of care for people with palliative care needs living in their own home: a systematic meta-review and narrative synthesis. Palliat Med.

[9] Finucane AM, O’Donnell H, Lugton J, Gibson-Watt T, Swenson C, Pagliari C (2021). Digital health interventions in palliative care: a systematic meta-review. NPJ Digit Med.

[10] Weaver MS, Neumann ML, Navaneethan H, Robinson JE, Hinds PS (2020). Human touch via Touchscreen: rural nurses’ experiential perspectives on Telehealth Use in Pediatric Hospice Care. J Pain Symptom Manag.

[11] Bradford NK, Young J, Armfield NR, Herbert A, Smith AC (2014). Home telehealth and paediatric palliative care: clinician perceptions of what is stopping us?. BMC Palliat Care.

[12] Aas E, Iversen T, Kaarboe O (2021). The Economic sustainability of the Norwegian Healthcare System.

[13] The Norwegian Directorate of Health. Palliative care for children and young people, National guidelines. 2016. https://www.helsedirektoratet.no/retningslinjer/palliasjon-til-barn-og-unge

[14] Ministry of Local Government and Regional Development. One digital public sector. Digital strategy for the public sector 2019–2025. In: Development MoLGaR, editor. 2019.

[15] Lundgren A, Randall L, Norlén G (2020). State of the Nordic Region 2020 -Wellbeing, health and digitalisation.

[16] Nick E, Nick E (2013). Purposeful sampling. Sampling and choosing cases in qualitative research: a Realist Approach.

[17] Holmen H. Gathering evidence for evidence-based health technology to support communication in homebased pediatric palliative care – CHIP homeTec Oslo: The Research Council of Norway; 2021. https://prosjektbanken.forskningsradet.no/en/project/FORISS/314850?Kilde=FORISS&distribution=Ar&chart=bar&calcType=funding&Sprak=no&sortBy=date&sortOrder=desc&resultCount=30&offset=30

[18] TSD (2024). Tjeneste for Sensitive Data. 2.0 ed.

[19] Brinkmann S, Kvale S (2018). Planning an interview study. Doing interviews. Qualitative Research Kit. Second ed.

[20] Brinkmann S, Kvale S. Interview variations. Doing Interviews. Qualitative Research Kit. Second ed. 55 City Road: 55 City Road: SAGE Publications Ltd; 2018. p. 73.

[21] Radford A, Kim JW, Xu T, Brockman G, McLeavey C, Sutskever I. Robust Speech Recognition via Large-Scale Weak Supervision. arXivorg. 2022.

[22] Braun V, Clarke V, Braun V (2022). Thematic analysis: a practical guide.

[23] Schröder J, Riiser K, Holmen H (2024). The needs of healthcare personnel who provide home-based pediatric palliative care: a mixed method systematic review. BMC Health Serv Res.

[24] Livetools AS. KnowMe Kongsberg 2024. https://www.knowme.no/

[25] Gauhe G, Cisneros REK, Ward J, Hohenschurz-Schmidt DJ (2023). Creatively adapting touch-based practices to the web Format during the COVID-19 pandemic: systematic review. J Med Internet Res.

[26] Caulfield BM, Donnelly SC (2013). What is Connected Health and why will it change your practice?. QJM.

[27] Delemere E, Gitonga I, Maguire R (2022). Utility, barriers and facilitators to the use of connected health to support families impacted by paediatric cancer: a qualitative analysis. Support Care Cancer.

[28] Pattichis CS, Panayides AS (2019). Connected health. Front Digit Health.

[29] Bush RA, Pérez A, Baum T, Etland C, Connelly CD (2018). A systematic review of the use of the electronic health record for patient identification, communication, and clinical support in palliative care. JAMIA Open.

[30] Meyer D, Kernebeck S, Busse TS, Ehlers J, Wager J, Zernikow B (2021). Electronic Health Records in Specialized Pediatric Palliative Care: a qualitative needs Assessment among professionals experienced and Inexperienced in Electronic Documentation. Children.

[31] Hancock S, Preston N, Jones H, Gadoud A (2019). Telehealth in palliative care is being described but not evaluated: a systematic review. BMC Palliat Care.

[32] World Health Organization. Global strategy on digital health 2020–2025. 2021. https://apps.who.int/iris/bitstream/handle/10665/344249/9789240020924-eng.pdf

[33] Winger A, Kvarme LG, Løyland B, Kristiansen C, Helseth S, Ravn IH (2020). Family experiences with palliative care for children at home: a systematic literature review. BMC Palliat Care.

[34] Holmen H, Winger A, Steindal SA, Riiser K, Castor C, Kvarme LG (2023). Patient-reported outcome measures in children, adolescents, and young adults with palliative care needs—a scoping review. BMC Palliat Care.

