# Supplementary material for: Healthcare personnel’s perspectives on health technology in home-based pediatric palliative care: a qualitative study
Source: BMC Palliat Care. 2024 May 29;23:137. doi: 10.1186/s12904-024-01464-w (PMC11134737; doi:10.1186/s12904-024-01464-w)
Supplement: Supplementary file 1 — Supplementary Material 1 [file 12904_2024_1464_MOESM1_ESM.docx]

# Supplementary material 1. Semistructured interview guide

| Interview questions | Further questions |
| --- | --- |
| 1. Please share your experiences and opinions about the use of health technology:  - when you meet the child and family  - when you collaborate with colleagues or others in the interdisciplinary team  - through your workday | Are the experiences mentioned true for the rest of you?  Are you able to give examples?  Can you explain more...? |
| 2. Please share your experiences and opinions about barriers to health technology. |  |
| 3. Please share your experiences and opinions about the limitations of health technology. |  |
| 4. If you could develop a digital solution, please share your experiences and opinions on what would improve your daily workday. |  |
| 5. Do you want to discuss or share other relevant issues? |  |
